# HEXACO, the Dark Triad, and Chat GPT: Who is willing to commit academic cheating?

**DOI:** 10.1016/j.heliyon.2023.e19909

**Published:** 2023-09-06

**Authors:** Tobias Greitemeyer, Andreas Kastenmüller

**Affiliations:** a University of Innsbruck, Austria; b University of Siegen, Germany

**Keywords:** Chat GPT, Academic cheating, HEXACO, Dark Triad

## Abstract

The rise in popularity of Chat GPT, an advanced language model that uses deep learning techniques to simulate human-like conversation, has raised concerns about its potential misuse, particularly in academic contexts. The present study (*N* = 283) explored the relationship between personality traits and the intention to use chatbot-generated texts for academic cheating. Among the HEXACO and Dark Triad traits, Honesty-Humility, Conscientiousness, Openness to Experience (all negative), Machiavellianism, narcissism, and psychopathy (all positive) were significant predictor variables. A multiple regression analysis showed that Honesty-Humility had the most robust association with the intention to use chatbot-generated texts for academic cheating. Further analyses on the facet level revealed that the fairness facet of Honesty-Humility was the most predictive, suggesting that individuals high in Honesty-Humility refrain from using chatbot-generated texts for academic cheating as they prioritize fairness over their own interests. Promoting Honesty-Humility and its fairness facet can be a valuable approach to promoting ethical behavior in academic and other contexts.

## 1 Introduction

Academic cheating has been a persistent problem in educational settings, and the rise of advanced language models, such as Chat GPT, raises concerns about the potential for these technologies to facilitate cheating behaviors. For example, the ability to generate high-quality text makes Chat GPT an attractive tool for students to complete their assignments with minimal effort. Hence, it is important to understand the factors that influence an individual's intention to use chatbot-generated texts without acknowledging the source as a form of academic cheating.

Personality traits have been found to be important predictors of a range of unethical and immoral behaviors, including cheating [1,2]. The current study investigates the relationship between personality traits and the intention to use chatbot-generated texts for academic cheating.^1^ As personality traits, we considered the HEXACO model as well as the Dark Triad. The HEXACO model comprises six broad personality dimensions, some of these traits have been found to be particularly relevant in understanding (un)ethical behavior (described in detail below). The Dark Triad encompasses three specific personality traits associated with self-centered and manipulative tendencies, and we thus reasoned that these traits are associated with a greater willingness to engage in cheating behaviors for personal gain.

### 1.1 Chat GPT

Chat GPT (Generative Pre-trained Transformer) is an advanced language model that uses deep learning techniques to simulate human-like conversation. It was released on November 30th, 2022. Within just four days of its launch, over one million users had started using the platform. By January 2023, the user base had grown exponentially to over 100 million users, making Chat GPT the most rapidly expanding consumer application in history [3]. Developed by OpenAI, Chat GPT is capable of generating highly coherent and contextually relevant text, making it a powerful tool for natural language processing tasks such as language translation, content generation, and conversational agents.

Chat GPT is able to generate text that is difficult to distinguish from human-generated text and has thus raised concerns about the potential for its misuse, particularly in academic contexts where it could be used to facilitate cheating behaviors. For example, students who utilize Chat GPT as a resource for their seminar papers without acknowledging its use may pose difficulties for educators in accurately evaluating the students' knowledge and writing proficiency. Furthermore, although students may potentially receive higher grades with less effort when using Chat GPT, they forego the opportunity to cultivate essential skills such as critical thinking and writing.

### 1.2 Personality traits and academic cheating

The present study explores the relationship between personality traits and university students’ willingness to use chatbot-generated texts for their seminar work without acknowledging the source (that is, committing academic cheating). While some personality models seek to encompass the full range of personality traits through a limited number of broad factors, other personality theories concentrate on specific traits. Here, we employ the HEXACO as a broad personality model and the Dark Triad as a narrow set of personality traits.

The HEXACO model of personality attempts to capture the fundamental dimensions of human personality [4,5]. It proposes that there are six major domains of personality, each of which is assessed in the HEXACO inventories by four facets, resulting in a total of 24 facets. The six domains are: Honesty-Humility, Emotionality, Extraversion, Agreeableness, Conscientiousness, and Openness to Experience. Individuals with high scores on Honesty-Humility avoid manipulating others for personal gain, follow rules, and are not motivated by material gain or social status. Individuals with high scores on Emotionality experience fear and anxiety and feel sentimental attachments. Individuals with high scores on Extraversion enjoy socializing and feel enthusiastic and energetic. Individuals with high scores on Agreeableness forgive and cooperate with others and are lenient in their judgments. Individuals with high scores on Conscientiousness are organized and work diligently. Individuals with high scores on Openness to Experience are curious and interested in unusual ideas or people.

The HEXACO model of personality is an extension of the Big Five, arguably the most dominant model in personality psychology. With the inclusion of Honesty-Humility as an additional factor, it emphasizes the role of ethical and moral aspects of personality, distinguishing it from the Big Five. Therefore, the HEXACO model, with its explicit focus on ethical dimensions and the inclusion of Honesty-Humility, provides a comprehensive and suitable framework for understanding the relationship between personality traits and the intention to use chatbot-generated texts for academic cheating.

In fact, among the HEXACO factors, research has consistently shown that Honesty-Humility is negatively related to various forms of immoral behavior. According to a recent meta-analysis [6], Honesty-Humility is strongly and negatively associated with various forms of unethical behavior, including lying, cheating, and stealing. Because Honesty-Humility exhibits the strongest relationship with immoral behavior compared to the other personality factors within the HEXACO model (see also [7]), we expected Honesty-Humility to be particularly predictive of using chatbot-generated texts.

Another meta-analysis [8] about the impact of the Big Five personality traits found that Conscientiousness was a negative predictor of academic dishonesty. Hence, we hypothesized that there would be a negative relationship between Conscientiousness and the use of chatbot-generated texts.

In contrast, we predicted Openness to Experience to be positively related to the use of chatbot-generated texts. We reasoned that individuals who score high on Openness to Experience are more likely to try out new tools (such as Chat GPT). On the other hand, Zettler et al. [6] found that Openness to Experience was (weakly) negatively associated with unethical behavior. Hence, we were less sure about how Openness to Experience would relate to the use of chatbot-generated texts.

We did not have any a priori expectations about the relationships between Emotionality, Extraversion, and Agreeableness and the use of chatbot-generated texts. However, we included these factors in our analysis for exploratory reasons.

In contrast to the HEXACO model, the Dark Triad is considered a narrow personality model. The Dark Triad is a cluster of three distinct personality traits: narcissism, Machiavellianism, and psychopathy [9]. Narcissism involves an exaggerated sense of self-importance and a need for admiration. Machiavellianism is characterized by a tendency to use manipulative tactics and deceptive strategies to achieve one's goals. Psychopathy includes characteristics such as callousness and unemotionality. While each trait has its unique characteristics, they also have some common cores in that all three traits are characterized by a strong focus on oneself and personal interests, a reduced capacity for empathy and concern for others, and the use of manipulative behavior to achieve one's goals or gain advantages [[10], [11], [12], [13]].

Some studies have shown positive links between all Dark Triad traits and academic misconduct [[14], [15], [16]]. However, other studies [17,18] found that academic cheating was significantly positively correlated with psychopathy and Machiavellianism, but not with narcissism. In another study [19], Machiavellianism and narcissism were positively associated with self-reported academic cheating behaviors, whereas psychopathy was not. Overall, we expected Machiavellianism to have the strongest association with the use of chatbot-generated texts among the Dark Triad traits, as individuals high in Machiavellianism are particularly prone to view cheating as a means to an end.

It should be noted that some scholars argue that the Dark Triad traits are primarily represented by the low end of the Honesty-Humility dimension [20,21]. They contend that there is substantial overlap in the shared variance between the Dark Triad traits and low Honesty-Humility, indicating that the Dark Triad traits offer limited additional predictive value beyond the inverse facet of Honesty-Humility. However, other research indicates that the Dark Triad traits are not merely the opposite end of the Honesty-Humility dimension and that they do offer valuable additional insights [[22], [23], [24]]. Hence, we investigated whether Machiavellianism, as well as narcissism and psychopathy, would predict the intention to use chatbot-generated texts beyond the HEXACO dimensions, particularly Honesty-Humility.

Some individuals may refrain from using chatbot-generated texts not because of ethical considerations but because of quality concerns. For example, we expected individuals with high Honesty-Humility to be less likely to use chatbot-generated texts due to their aversion to breaking rules for personal gain. Alternatively, they may perceive chatbot-generated texts to be of low quality, which could discourage their usage. To differentiate between these possibilities, we included a measure of perceived chatbot text quality in our analyses.

### 1.3 The present research

The present study was carried out to examine which broad (HEXACO) and narrow (Dark Triad) personality traits are associated with a greater willingness to use chatbot-generated texts. Participants were asked about their willingness to use texts generated by chatbots for their seminar papers, without disclosing the involvement of AI in their creation.

We pre-registered (https://aspredicted.org/N12_3K7) the following hypotheses:

H1 Honesty-Humility is negatively related to the intention to use chatbot-generated texts.

H2 Conscientiousness is negatively related to the intention to use chatbot-generated texts.

H3 Openness to Experience is positively related to the intention to use chatbot-generated texts.

H4 Machiavellianism is positively related to the intention to use chatbot-generated texts.

To test our hypotheses, we conducted correlation analyses. In ancillary analyses, we report on the relationship between the intention to use chatbot-generated texts and the 24 HEXACO facets. A regression analysis aimed to identify the personality traits that predict the intention to use chatbot-generated texts, while controlling for the influence of the other personality traits. Finally, to disentangle whether participants do not use chatbot-generated texts because they do not endorse dishonesty or because they are concerned about the quality of their seminar papers, we report on partial correlations (controlling for the perceived quality of chatbot texts). These analyses were conducted using IBM SPSS 25.

The study received ethical approval from the Internal Review Board for Ethical Questions by the Scientific Ethical Committee of our university (Certificate of good standing, 90/2022). The data are publicly available at: https://osf.io/quwh6/?view_only=27a2fb43921c4e56b6e29f0a3b459ae0.

## 2 Method

### 2.1 Participants

Participants were invited via an Austrian university mailing list. At the beginning of the survey, the participants were informed about the purpose of the study and voluntary consent was obtained. Two hundred and eighty-three students completed the questionnaire (186 women, 94 men, 3 diverse, mean age: 25.0, *SD* = 7.75). There were some gender differences on personality traits (e.g., women had lower scores on the Dark Triad traits than men), but participant sex was not related to the intention to use chatbot-generated texts. Hence, this variable is not considered further. Data collection took place in February 2023. A sensitivity analysis showed that with this sample size, the study had 80% statistical power to detect a correlation of *r* ≥ 0.16, corresponding to small to medium sized (and larger) effects. Among all participants, three cash prizes of 50 Euros were raffled.

### 2.2 Procedure and measures

After participants provided their demographic data, they learned the following:

“Recently, new artificial intelligence (AI) tools (such as Chat GPT) have been developed that generate artificial text. By using algorithms and accessing a huge database, texts are generated that can be used by anyone. For example, students could have their seminar papers (or parts of them) written by the computer.

As these texts are generated artificially, they are original and do not draw upon any pre-existing sources. When creating the texts, linguistic models are used that use probabilities to determine which word could follow the previous one. Therefore, the generated texts are not perfect, but they are usually so well thought-out that it is difficult to tell whether they were created by a human or a computer.

Below we present a text developed by the Chat GPT tool. We instructed the tool to generate a scientific text on measures to combat inflation, providing no further instructions beyond this. The content of the following text has been created exclusively by the Chat GPT tool.

Measures to combat inflation include both monetary and fiscal policy instruments. Monetary measures involve increasing the central bank's interest rates to raise borrowing costs and thus reduce demand for consumer goods and investments. Fiscal policy measures involve a restrictive fiscal policy, where the government cuts its spending and/or increases taxes to reduce demand. Central banks can also intervene to influence the exchange rate of their currency and thus reduce inflation. A strict monetary policy, where the central bank limits the money supply, can also help to suppress inflation. However, it should be noted that these measures may have negative effects on economic growth and that the choice of appropriate measures depends on the type of inflation and the circumstances of the respective country."

To evaluate the perceived quality of chatbot-generated texts, participants were asked: “To what extent do you find AI-generated texts readable?“, “How accurate do you consider the content of such AI texts to be?“, and “How suitable are such AI texts as a part of a seminar paper?“. The scale was from 1 (*very little*) to 7 (*very*). To create an index of the perceived quality of chatbot texts, responses were averaged (α = 0.63).

Participants were then told:

“The use of AI-generated texts without acknowledging its source is considered an act of academic misconduct. At the University of Innsbruck, for instance, students are required to independently author their academic work and to only use the sources and aids specified. For the following questions, we are interested in your willingness to use AI-generated texts without acknowledging its source (without acknowledging its source in bold). Please note that all responses (including those to other questions) will be treated confidentially and no personal conclusions can be drawn.”

Next, participants were presented with two statements: “I refuse to use AI-generated texts for my seminar papers.” (recoded, *M* = 3.02, *SD* = 2.05) and “I might consider using AI-generated texts for my seminar papers in the future.” (*M* = 2.93, *SD* = 1.89). Participants rated their level of agreement with each statement on a scale from 1 (*do not agree at all*) to 7 (*agree completely*). Participants were also asked: “What percentage of AI-generated text can you imagine using in one of your seminar papers?” (*M* = 18.5, *SD* = 20.1). These three responses were z-standardized and averaged to create an index of the intention to use chatbot-generated texts in one's seminar papers without acknowledging its source (α = 0.86).

Participants then responded to the 100-item HEXACO-Personality Inventory [25]. The scale for all items was 1 from to 5. Scale reliabilities were: Honesty-Humility: α = 0.83, Emotionality: α = 0.83, Extraversion: α = 0.87, Agreeableness: α = 0.82, Conscientiousness: α = 0.83, Openness to Experience: α = 0.75. Finally, the Short Dark Triad [26] was employed to assess narcissism (α = 0.74), Machiavellianism (α = 0.80), and psychopathy (α = 0.70). For each Dark Triad trait, there were nine items on a 1- to 5-point scale.

## 3 Results

Descriptive statistics and intercorrelations of all measures are shown in Table 1. The data confirmed H1 and H2 that Honesty-Humility and Conscientiousness had a negative relationship with the intention to use chatbot-generated texts. Unexpectedly, in contrast to H3, Openness to Experience had a negative association with the intention to use chatbot-generated texts. Supporting H4, Machiavellianism was positively related to the intention to use chatbot-generated texts. Furthermore, both narcissism and psychopathy were positively related to the intention to use chatbot-generated texts.

**Table 1:** Means, standard deviations, and bivariate correlations.

	*M*	*SD*	1	2	3	4	5	6	7	8	9	10
1. Intention	−0.01	0.88										
2. Perceived quality	4.83	1.05	.30***									
3. Honesty-Humility	3.53	0.60	−.27***	−.07								
4. Emotionality	3.28	0.61	−.11	−.25***	.02							
5. Extraversion	3.26	0.63	.04	.14*	−.05	−.20**						
6. Agreeableness	3.11	0.55	.02	.05	.28***	−.23***	.07					
7. Conscientiousness	3.55	0.55	−.15*	−.08	.05	.19**	.20**	−.14*				
8. Openness to Experience	3.52	0.52	−.12*	.11	.24***	−.03	.11	.12*	.07			
9. Narcissism	2.51	0.66	.20**	.09	−.34***	−.23***	.48***	−.26***	.08	.07		
10. Machiavellianism	2.72	0.73	.13*	.07	−.62***	−.09	−.18**	−.38***	−.00	−.20**	.27***	
11. Psychopathy	2.00	0.59	.24***	.15	−.46***	−.25***	.05	−.24***	−.29***	−.01	.42***	.48***

*p < .05, **p < .01, ***p < .001.

**Fig. 1:**
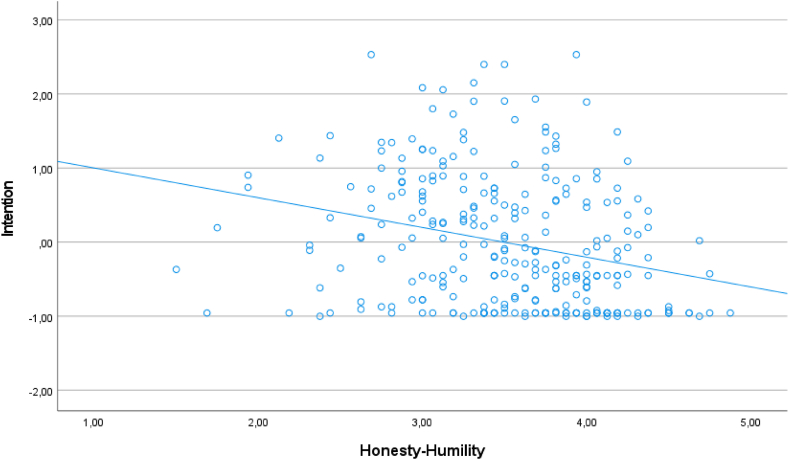
A scatterplot of the correlation between Honesty-Humility and the intention to use chatbot-generated texts for academic cheating.

Next, a multiple regression was performed on the data, using the HEXACO traits and the Dark Triad traits as predictors for the intention to use chatbot-generated texts. The overall regression was significant, *F*(9, 282) = 4.64, *p* < .001, *R*^2^ = 0.13. Among all predictor variables, only Honesty-Humility received a significant regression weight, β = −0.23, *p* = .003 (see Fig. 1 for a scatterplot of the relation between Honesty-Humility and the intention to use chatbot-generated texts).

Of the HEXACO facets, fairness, *r*(283) = −0.32, *p* < .001, greed avoidance, *r*(283) = −0.14, *p* = .019, modesty, *r*(283) = −0.17, *p* = .004, anxiety, *r*(283) = −0.13, *p* = .028, diligence, *r*(283) = −0.19, *p* = .002, perfectionism, *r*(283) = −0.17, *p* = .005, prudence, *r*(283) = −0.12, *p* = .045, and creativity, *r*(283) = −0.19, *p* = .001, were significantly related to the intention to use chatbot-generated texts. A multiple regression was performed next, with all the HEXACO facets as predictors for the intention to use chatbot-generated texts. The overall regression was significant, *F*(24, 258) = 3.19, *p* < .001, *R*^2^ = 0.23. Fairness, β = −0.30, *p* = .003, organization, β = 0.16, *p* = .025, diligence, β = −0.17, *p* = .036, and creativity, β = −0.21, *p* = .002, received significant regression weights.

Not surprisingly, the intention to use chatbot-generated texts was positively related to their perceived quality (see Table 1). It was therefore examined whether the relationships between personality traits and the intention to use chatbot-generated texts remain significant when controlling for their perceived quality. Partial correlations, controlling for the perceived quality of chatbot texts, showed that the relationships between the intention to use chatbot-generated texts and Honesty-Humility, *r*(280) = −0.26, *p* < .001, Conscientiousness, *r*(280) = −0.13, *p* = .026, and Openness to Experience, *r*(280) = −0.16, *p* = .008, remained significant. In contrast, the relationship between the intention to use chatbot-generated texts and Machiavellianism was no longer significant, *r*(280) = 0.12, *p* = .052, while it remained significant for narcissism, *r*(280) = 0.18, *p* = .003, and psychopathy, *r*(280) = 0.21, *p* = .001.

## 4 Discussion

Academic dishonesty is a prevalent issue within educational environments, and the emergence of advanced language models like Chat GPT has sparked concerns regarding their potential role in facilitating cheating behaviors. The aim of the present study was to explore the role of personality traits in predicting an individual's intention to utilize chatbot-generated texts as a means to commit academic fraud. By examining a diverse range of personality traits, we aimed to determine whether certain traits were associated with a higher propensity to engage in this form of fraudulent behavior.

The results confirmed the hypotheses that Honesty-Humility and Conscientiousness were negatively related to the intention to use chatbot-generated texts and are consistent with previous research on academic dishonesty showing that individuals with high levels of Honesty-Humility and Conscientiousness are less likely to engage in cheating behaviors [6,8].

Further analysis on the facet level revealed that several facets of Honesty-Humility and Conscientiousness were significantly negatively associated with the intention to use chatbot-generated texts. Among these, the fairness facet of Honesty-Humility and the diligence facet of Conscientiousness were the most predictive. Hence, Honesty-Humility appears to be negatively associated with academic cheating because individuals who prioritize fairness over their own interests are less likely to cheat. Likewise, the negative relationship between Conscientiousness and the intention to use chatbot-generated texts is due to high scorers on diligence having a strong work ethic and being willing to exert themselves.

Contrary to H3, which suggested a positive association between Openness to Experience and the intention to use chatbot-generated texts, the relationship was negative (which is a replication of Zettler et al. [6]). We initially reasoned that individuals who are more open to new experiences are more likely to experiment with chatbot technology, out of curiosity or a desire to try new things. Further analysis on the facet level revealed that creativity was negatively associated with the intention to use chatbot-generated texts. Individuals who score high on this scale tend to actively seek new solutions to problems, whereas low scorers have little inclination for original thought. Hence, it appears that individuals who score low on Openness to Experience are more likely to rely on chatbots, as they are less inclined to seek out creative or innovative solutions. In contrast, individuals who score high on Openness to Experience prefer to tackle challenges with their own original ideas. It might be that individuals with high levels on Openness to Experience are more inclined to try out tools such as Chat GPTwhen the use does not involveacademic cheating.

Consistent with H4, Machiavellianism was positively related to the intention to use chatbot-generated texts, suggesting that individuals who are manipulative and strategic are more likely to use chatbots to achieve their goals and to gain an advantage. Additionally, both narcissism and psychopathy were positively related to the intention to use chatbot-generated texts, indicating that individuals who are self-focused and unemotional are more inclined to use chatbot technology for personal gain. Some previous studies have shown that Machiavellianism, but not psychopathy [19] or narcissism [17,18], is related to academic cheating. The present findings corroborate other previous work showing that all Dark Triad traits are positive predictors of academic misconduct [[14], [15], [16]]. Overall, and contrary to our expectation that Machiavellianism would exhibit a stronger relationship with academic cheating than the two other Dark Triad traits, it appears that all traits within the Dark Triad share the common dark core that individuals high in these traits prioritize their own interests and personal gain [27,28] over ethical considerations, which leads them to engage in academic cheating.

Finally, the study examined whether the relationships between personality traits and the intention to use chatbot-generated texts remained significant when controlling for the perceived quality of chatbot texts. As most of the relationships remained significant (with the exception of Machiavellianism), it appears that the impact of personality on the intention to use chatbot-generated texts is not primarily driven by concerns about the quality of chatbot texts, but rather by adherence (or lack thereof) to ethical considerations.

### 4.1 Practical and theoretical implications

By examining the relationship between personality traits and the intention to use chatbot-generated texts for academic cheating, our study sheds light on the factors that contribute to this unethical behavior. With the increasing popularity of AI models, there are worries about their application in academic settings, specifically in relation to academic cheating. Our findings highlight the importance of considering personality traits when addressing the potential misuse of advanced language models and provide insights that can inform interventions and strategies aimed at promoting integrity and ethical conduct in academic settings. By promoting Honesty-Humility, particularly its fairness facet, ethical behavior in academic and other contexts can be encouraged.

Theoretical implications of this study emerge from the finding that, when all predictor variables were simultaneously taken into account, only Honesty-Humility demonstrated a significant predictive effect on the intention to utilize chatbot-generated texts. This finding aligns with previous research that Honesty-Humility is more strongly associated with immoral behavior compared to other personality factors within the HEXACO model [6] and it emphasizes the advantage of using the HEXACO model instead of the traditional Big Five model in understanding the role of personality in predicting unethical behavior. By incorporating the HEXACO model, researchers can gain a deeper understanding of who is willing (and who is unwilling) to engage in unethical actions.

It also adds to the ongoing debate about whether the Dark Triad traits are fully represented by the Honesty-Humility dimension of the HEXACO model. Some scholars have argued that the common variance of the Dark Triad is nearly redundant with low Honesty-Humility, indicating that the Dark Triad traits offer little additional predictive validity beyond the inverse facet of Honesty-Humility [20,21], whereas other research has shown that the Dark Triad traits are not merely the opposite end of the Honesty-Humility dimension [[22], [23], [24]]. In terms of the intention to use chatbots for academic misconduct, it appears that the broad Honesty-Humility trait is sufficient and even more predictive than the narrower Dark Triad traits.

Recently, the Dark Triad has been extended to the Dark Tetrad, adding everyday sadism as a fourth trait [29,30]. Everyday sadism is characterized by the pleasure that is derived from harming others [31]. Future research may investigate whether everyday sadism, similar to narcissism, Machiavellianism, and psychopathy, is positively correlated with the inclination to employ chatbots for academic misconduct. Prior research has shown everyday sadism, and the Dark Tetrad in general, to be positively related to cheating [32]. It is noteworthy that this study also revealed that the relationship between the Dark Tetrad and cheating was fully accounted for by Honesty-Humility, which further suggests that Honesty-Humility is a better predictor of cheating behavior than dark personality traits.

### 4.2 Limitations and future research

A limitation is that this study was conducted at a single university, which does not allow strong conclusions about the generalizability of the findings. Additionally, although the sample size of 283 participants was sufficient to achieve adequate statistical power, it is worth noting that a larger sample could have yielded more robust and generalizable findings. Overall, the sample used in our study might have specific demographic, cultural, or contextual factors that limit the generalizability of the results to a broader population.

It should be also kept in mind that the present findings are based on intentions to use chatbot-generated texts, rather than actual behavior. Given that Chat GPT was only recently launched, we thought it unlikely that students already had used them for their seminar papers and thus we did not assess actual use. Nevertheless, there may be differences between what people say they intend to do and what they actually do. Therefore, future research should explore whether the same personality factors predict actual behavior related to the use of chatbots for academic misconduct.

A further limitation is the potential influence of social desirability bias on self-reported measures of personality traits [33,34] and the intention to commit academic cheating. Participants may have provided responses that they believed were socially acceptable, rather than their true thoughts or behavioral inclinations. In this regard, please note that the correlations between the various HEXACO scales were low. If social desirability had a significant impact, we would expect to observe strong correlations among these scales. This indicates that the responses are unlikely to be heavily biased by the respondents' tendency to present themselves in a socially desirable manner.

Another limitation of the present study is its cross-sectional design, which limits the ability to establish causal relationships between variables. Therefore, it is important to interpret the findings cautiously, as they only provide information about the associations between personality and the intention to use chatbot-generated texts and do not allow for conclusions about the direction of the relationships. Future research may employ a longitudinal design, which would provide more robust evidence regarding the temporal relationships between personality traits and the intention to use chatbot-generated texts.

## 5 Conclusions

The findings of this study hold significant implications in understanding the factors that contribute to the intention to utilize chatbot technology for academic cheating. As the popularity and usage of Chat GPT and other AI language models are expected to grow in the future, it becomes crucial to recognize both the opportunities they present and the potential risks they entail.

By illuminating the personality traits of individuals who are inclined to employ AI language models like Chat GPT for academic cheating, this study contributes valuable insights to the field. These insights can inform the development and implementation of preventive measures aimed at curbing the misuse of such technologies and promoting academic integrity.

As the educational landscape continues to evolve, it is imperative to proactively address the challenges posed by technological advancements. Understanding the determinants of an individual's willingness or reluctance to engage in academic cheating with the help of AI language models assists in formulating strategies to mitigate these risks effectively. By considering the findings of this study, educational institutions, policymakers, and relevant stakeholders can develop interventions, guidelines, and educational programs to raise awareness about the responsible use of AI language models, foster a culture of academic integrity, and discourage the misuse of these technologies for dishonest purposes.

## Author contribution statement

Tobias Greitemeyer: Conceived and designed the experiments; Performed the experiments; Analyzed and interpreted the data; Contributed reagents, materials, analysis tools or data; Wrote the paper.

Andreas Kastenmüller: Conceived and designed the experiments; Performed the experiments; Contributed reagents, materials, analysis tools or data.

## Data availability statement

Data associated with this study has been deposited at https://osf.io/quwh6/?view_only=27a2fb43921c4e56b6e29f0a3b459ae0.

## Declaration of competing interest

The authors declare that they have no known competing financial interests or personal relationships that could have appeared to influence the work reported in this paper.

## Footnotes

1 For the sake of brevity, in the following we will use the term “use of chatbot-generated texts” to refer specifically to using them for academic cheating purposes.
