# Supplementary material for: HEXACO, the Dark Triad, and Chat GPT: Who is willing to commit academic cheating?
Source: Heliyon. 2023 Sep 6;9(9):e19909. doi: 10.1016/j.heliyon.2023.e19909 (PMC10559323; doi:10.1016/j.heliyon.2023.e19909)
Supplement: Multimedia component 1 [file mmc1.pdf]

KI_Einstellung → base 08.03.2023, 11:10

Seite 01

TX04

Herzlich Willkommen!

Vielen Dank für Ihr Interesse an dieser Studie zu Einstellungen gegenüber künstlicher Intelligenz.

Teilnehmen können alle Personen, die aktuell an einer Universität eingeschrieben sind. Ein Mindestalter von 18 Jahren ist nötig. Selbstverständlich werden alle Bestimmungen des Datenschutzes eingehalten: Ihre Angaben werden vollständig anonymisiert und dienen ausschließlich dem wissenschaftlichen Zweck. Es können keinen Rückschlüsse auf Einzelpersonen oder Organisationen gezogen werden. Erhobene Daten werden niemals an Dritte weitergeben. Ihre Teilnahme ist freiwillig. Sie können die Befragung zu jeder Zeit ohne Begründung abbrechen. Durch die weitere Teilnahme bestätigen Sie Ihr Einverständnis, dass die Daten eventuell für wissenschaftliche Publikationen verwendet werden dürfen.

Wenn Sie den Fragebogen vollständig ausfüllen, laden wir Sie herzlich ein, an unserem Gewinnspiel mitzumachen: Unter den
Teilnehmenden werden **3 x 50€** verlost - als kleines Dankeschön für Ihre Unterstützung. Ihre E-Mail-Adresse wird im Rahmen des Gewinnspiels getrennt gespeichert, es können keine Rückschlüsse auf Ihre Angaben in der Umfrage gezogen werden.

Seite 02

DE01

1. Geschlecht:

- ○ weiblich
- ○ männlich
- ○ divers

DE02

2. Alter:

DE03

3. Studienfach:

Seite 03

TX01

In der letzten Zeit sind neue KI-Tools (wie z.B. Chat GPT) entwickelt worden, die beispielsweise künstliche Texte generieren. Durch den Einsatz von Algorithmen und Zugriff auf eine riesige Datenbasis werden Texte generiert, die von jedem genutzt werden können. Beispielsweise könnten Studierende sich Seminararbeiten (bzw. Teile von diesen) vom Computer schreiben lassen.

Diese künstlich generierten Texte werden nirgendwo abgeschrieben, der Text ist also nicht plagiiert. Beim Erstellen der Texte werden sprachliche Modelle verwendet, die mithilfe von Wahrscheinlichkeiten ermitteln, welches Wort auf das vorige folgen könnte. Von daher sind die generierten Texte nicht perfekt, jedoch zumeist so durchdacht, dass kaum nachzuweisen ist, ob die Texte durch einen Menschen oder durch den Computer erstellt wurden.

Seite 04

TX05

Beispiel eines von Chat GPT künstlich erzeugten Textes

Im Folgenden präsentieren wir Ihnen einen Text, den das Tool Chat GPT entwickelt hat. Wir hatten das Tool beauftragt, einen wissenschaftlichen Text zu Maßnahmen gegen Inflation zu verfassen. Außer dieser Vorgabe haben wir keine weiteren Instruktionen gegeben. Der Inhalt des folgenden Textes ist ausschließlich durch das Tool Chat GPT erstellt worden.

„Maßnahmen zur Bekämpfung von Inflation umfassen sowohl monetäre als auch fiskalpolitische Instrumente. Monetäre Maßnahmen beinhalten die Erhöhung der Leitzinsen durch die Zentralbank, um die Kreditkosten zu erhöhen und somit die Nachfrage nach Konsumgütern und Investitionen zu reduzieren. Fiskalpolitische Maßnahmen beinhalten eine restriktive Fiskalpolitik, bei der die Regierung ihre Ausgaben kürzt und/oder die Steuern erhöht, um die Nachfrage zu verringern. Zentralbanken können auch intervenieren, um den Wechselkurs ihrer Währung zu beeinflussen und damit die Inflation zu reduzieren. Eine strikte Geldpolitik, bei der die Zentralbank die Geldmenge begrenzt, kann ebenfalls dazu beitragen, die Inflation zu unterdrücken. Es ist jedoch zu beachten, dass diese Maßnahmen möglicherweise negative Auswirkungen auf das Wirtschaftswachstum haben können und dass die Wahl der geeigneten Maßnahmen von der Art der Inflation und den Umständen des jeweiligen Landes abhängt.“

Seite 05

F101

	1 – sehr wenig						7 -sehr
Als wie hoch schätzen Sie die Lesbarkeit solcher KI-Texte ein?	○	○	○	○	○	○	○
Als wie inhaltlich korrekt empfinden Sie solche KI Texte?	○	○	○	○	○	○	○
Wie gut sind solche KI Texte als Teil einer Seminararbeit geeignet?	○	○	○	○	○	○	○

Seite 06

TX03

Das Verwenden von KI-Texten ohne entsprechende Kennzeichnung stellt ein wissenschaftliches Fehlverhalten dar. So wird an der Universität Innsbruck verlangt, dass man wissenschaftliche Arbeiten selbständig verfasst und dass man keine anderen als die angegebenen Quellen und Hilfsmittel verwendet hat.

Bei den folgenden Fragen würde uns interessieren, inwiefern Sie KI-Texte **ohne Kennzeichnung** verwenden würden. Ihre Angaben helfen uns dabei, besser einschätzen zu können, wie man das Thema Chat GPT und ähnliche KI-Software in der universitären Lehre aufgreifen sollte. Selbstverständlich werden diese (und alle Ihrer anderen) Angaben vollständig anonym behandelt und es sind keine Rückschlüsse auf Ihre Person möglich):

F103

0% 100%

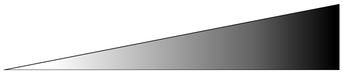

Welchen Anteil an KI-Text an der gesamten Arbeit können Sie sich vorstellen in einer ihrer Seminararbeiten zu verwenden?

F102

	stimme gar nicht zu						stimme voll zu
Ich lehne es ab, KI-Texte für meine Seminararbeiten zu verwenden.	○	○	○	○	○	○	○
Ich werde eventuell in der Zukunft KI-Texte für meine Seminararbeiten verwenden.	○	○	○	○	○	○	○

Seite 07

TX02

Auf den folgenden Seiten finden Sie eine Liste mit Aussagen, die mehr oder weniger auf Sie zutreffen können. Es gibt **keine** richtigen oder falschen Antworten. Bitte geben Sie an, wie sehr Sie den einzelnen Aussagen zustimmen oder sie ablehnen.

Seite 08

PE01

	starke Ablehnung	Ablehnung	Neutral	Zustimmung	Starke Zustimmung
Der Besuch einer Kunstausstellung würde mich ziemlich langweilen.	○	○	○	○	○
Ich putze mein Büro oder zuhause ziemlich oft.	○	○	○	○	○
Ich habe selten Wut im Bauch, nicht mal gegen Leute, die mich sehr ungerecht behandelt haben.	○	○	○	○	○
Im Allgemeinen bin ich mit mir ziemlich zufrieden.	○	○	○	○	○
Ich hätte Angst, wenn ich bei schlechten Wetterbedingungen verreisen müsste.	○	○	○	○	○
Wenn ich von einer Person, die ich nicht mag, etwas will, verhalte ich mich dieser Person gegenüber sehr nett um es zu bekommen.	○	○	○	○	○
Ich bin daran interessiert, etwas über die Geschichte und Politik anderer Länder zu lernen.	○	○	○	○	○
Wenn ich arbeite, setze ich mir oft ehrgeizige Ziele.	○	○	○	○	○
Andere sagen mir manchmal, dass ich zu kritisch gegenüber anderen bin.	○	○	○	○	○
Bei Gruppentreffen sage ich nur selten meine Meinung.	○	○	○	○	○
Ich kann manchmal nichts dagegen machen, dass ich mir über kleine Dinge Sorgen mache.	○	○	○	○	○
Wenn ich wüsste, dass ich niemals erwischt werde, wäre ich bereit, eine Million zu stehlen.	○	○	○	○	○
Ich wünsche mir einen Beruf, der Routine verlangt, anstatt einen, der Kreativität fordert.	○	○	○	○	○

Oft kontrolliere ich meine Arbeit mehrfach, um alle Fehler zu finden.	○	○	○	○	○
Andere sagen mir manchmal, dass ich zu dickköpfig bin.	○	○	○	○	○
Ich vermeide es, mit anderen Leuten Small Talk zu halten.	○	○	○	○	○
Wenn ich wegen einer schmerzvollen Erfahrung leide, brauche ich jemanden, der mich tröstet.	○	○	○	○	○
Viel Geld zu haben ist nicht besonders wichtig für mich.	○	○	○	○	○
Ich denke, dass es Zeitverschwendung ist, radikalen Ideen Aufmerksamkeit zu schenken.	○	○	○	○	○
Ich treffe Entscheidungen eher aus dem Bauch heraus als durch sorgfältiges Nachdenken.	○	○	○	○	○

Seite 09

PE02

	starke Ablehnung	Ablehnung	Neutral	Zustimmung	Starke Zustimmung
Andere halten mich für jähzornig.	○	○	○	○	○
Ich bin fast immer voller Energie.	○	○	○	○	○
Ich könnte weinen, wenn ich andere Personen sehe, die weinen.	○	○	○	○	○
Ich bin eine ganz normale Person, die nicht besser ist als andere.	○	○	○	○	○
Ich würde meine Zeit nicht damit verbringen, einen Gedichtband zu lesen.	○	○	○	○	○
Ich plane im Voraus und organisiere, damit in letzter Minute kein Zeitdruck aufkommt.	○	○	○	○	○
Meine Einstellung gegenüber Personen, die mich schlecht behandelt haben, ist „vergeben und vergessen“.	○	○	○	○	○
Ich glaube, dass die meisten Menschen einige Aspekte meines Charakters mögen.	○	○	○	○	○
Es stört mich nicht, Arbeiten zu erledigen, die gefährlich sind.	○	○	○	○	○
Ich würde keine Schmeicheleien benutzen, um eine Gehaltserhöhung zu bekommen oder befördert zu werden, auch wenn ich wüsste, dass es erfolgreich wäre.	○	○	○	○	○
Ich mag es, Landkarten von anderen Orten zu betrachten.	○	○	○	○	○
Ich treibe mich oft selbst sehr stark an, wenn ich versuche, ein Ziel zu erreichen.	○	○	○	○	○
Ich akzeptiere im Allgemeinen die Schwächen anderer, ohne mich darüber zu beschweren.	○	○	○	○	○

In sozialen Situationen bin ich gewöhnlich der, der den ersten Schritt macht.	○	○	○	○	○
Ich mache mir viel weniger Sorgen als die meisten Leute.	○	○	○	○	○
Ich würde in Versuchung geraten, Diebesgut zu kaufen, wenn ich knapp bei Kasse wäre.	○	○	○	○	○
Ich würde es genießen, ein Kunstwerk zu schaffen, etwa einen Roman, ein Lied oder ein Gemälde.	○	○	○	○	○
Wenn ich an irgendetwas arbeite, beachte ich kleine Details nicht allzu sehr.	○	○	○	○	○
Ich bin gewöhnlich ziemlich flexibel in meinen Ansichten, wenn andere Leute mir nicht zustimmen.	○	○	○	○	○
Ich genieße es, viele Leute um mich herum zu haben, mit denen ich reden kann.	○	○	○	○	○

Seite 10

PE03

	starke Ablehnung	Ablehnung	Neutral	Zustimmung	starke Zustimmung
Ich kann mit schwierigen Situationen umgehen, ohne dass ich emotionale Unterstützung von irgendjemandem brauche.	○	○	○	○	○
Ich würde gerne in einer sehr teuren, angesehenen Nachbarschaft wohnen.	○	○	○	○	○
Ich mag Leute, die unkonventionelle Ideen haben.	○	○	○	○	○
Ich mache viele Fehler, weil ich nicht nachdenke, bevor ich handele.	○	○	○	○	○
Ich werde selten wütend, selbst wenn andere mich ziemlich schlecht behandeln.	○	○	○	○	○
An den meisten Tagen bin ich fröhlich und optimistisch.	○	○	○	○	○
Wenn jemand, den ich gut kenne, unglücklich ist, kann ich den Schmerz dieser Person fast selber spüren.	○	○	○	○	○
Ich will nicht, dass andere Leute mich behandeln, als ob ich ihnen überlegen sei.	○	○	○	○	○
Wenn ich die Gelegenheit dazu hätte, würde ich gerne ein Konzert mit klassischer Musik besuchen.	○	○	○	○	○
Andere machen oft mit mir zusammen Witze über die Unordentlichkeit meines Zimmers oder Schreibtisches.	○	○	○	○	○
Wenn mich jemand einmal betrogen hat, werde ich dieser Person gegenüber immer misstrauisch bleiben.	○	○	○	○	○
Ich bin der Meinung, dass ich nicht beliebt bin.	○	○	○	○	○
Wenn es um körperliche Gefahren geht, bin ich sehr ängstlich.	○	○	○	○	○

Wenn ich von jemandem etwas will, lache ich auch noch über dessen schlechteste Witze.	○	○	○	○	○
Ich wäre von einem Buch über die Geschichte der Wissenschaft und Technik sehr gelangweilt.	○	○	○	○	○
Wenn ich mir ein Ziel setze, gebe ich oft auf, bevor ich es erreicht habe.	○	○	○	○	○
Ich neige dazu, nachsichtig zu sein, wenn ich andere beurteile.	○	○	○	○	○
Wenn ich in einer Gruppe von Leuten bin, bin ich oft derjenige, der im Namen der Gruppe spricht.	○	○	○	○	○
Ich habe selten, wenn überhaupt, Schlafprobleme durch Stress oder Angst.	○	○	○	○	○
Ich würde niemals Bestechungsgeld annehmen, auch wenn es sehr viel wäre.	○	○	○	○	○

Seite 11

PE04

	starke Ablehnung	Ablehnung	neutral	Zustimmung	starke Zustimmung
Man hat mir schon oft gesagt, dass ich eine gute Vorstellungskraft habe.	○	○	○	○	○
Ich versuche immer, fehlerfrei zu arbeiten, auch wenn es Zeit kostet.	○	○	○	○	○
Wenn mir andere sagen, dass ich falsch liege, ist meine erste Reaktion, mit ihnen zu streiten.	○	○	○	○	○
Ich ziehe Berufe, in denen man sich aktiv mit anderen Menschen auseinandersetzt solchen vor, in denen man alleine arbeitet.	○	○	○	○	○
Wenn ich mir um irgendetwas Sorgen mache, will ich meine Sorgen mit einer anderen Person teilen.	○	○	○	○	○
Ich würde gerne dabei gesehen werden, wie ich in einem sehr teuren Auto herumfahre.	○	○	○	○	○
Ich halte mich selber für eine etwas exzentrische Person.	○	○	○	○	○
Ich lasse nicht zu, dass meine Impulse mein Verhalten dominieren.	○	○	○	○	○
Die meisten Leute werden schneller ärgerlich als ich.	○	○	○	○	○
Andere sagen mir oft, dass ich versuchen sollte, etwas fröhlicher zu sein.	○	○	○	○	○
Ich fühle starke Emotionen, wenn jemand, der mir nahe steht, für eine längere Zeit weggeht.	○	○	○	○	○
Ich denke, dass ich mehr Respekt verdiene als ein durchschnittlicher Mensch.	○	○	○	○	○
Manchmal mag ich es, einfach nur dem Wind zuzusehen, wie er durch die Bäume bläst.	○	○	○	○	○

Wenn ich arbeite, habe ich manchmal Schwierigkeiten, weil ich unorganisiert bin.	○	○	○	○	○
Es fällt mir schwer, jemandem vollkommen zu vergeben, der mir etwas Gemeines angetan hat.	○	○	○	○	○
Manchmal habe ich den Eindruck, dass ich wertlos bin.	○	○	○	○	○
Selbst in einem Notfall würde ich nicht in Panik geraten.	○	○	○	○	○
Ich würde nicht vortäuschen, jemanden zu mögen, nur um diese Person dazu zu bringen, mir Gefälligkeiten zu erweisen.	○	○	○	○	○
Ich habe es noch nie wirklich gemocht, eine Enzyklopädie durchzublättern.	○	○	○	○	○
Ich arbeite nur so viel wie nötig, um gerade so durchzukommen.	○	○	○	○	○

Seite 12

PE05

	starke Ablehnung	Ablehnung	neutral	Zustimmung	starke Zustimmung
Selbst wenn Leute viele Fehler machen, sage ich nur selten etwas Negatives.	○	○	○	○	○
Ich fühle mich nicht ganz behaglich wenn ich vor einer Gruppe von Leuten spreche.	○	○	○	○	○
Ich werde sehr unruhig, wenn ich auf eine wichtige Entscheidung warte.	○	○	○	○	○
Ich würde in die Versuchung geraten, Falschgeld zu benutzen, wenn ich sicher sein könnte, damit durchzukommen.	○	○	○	○	○
Ich halte mich nicht für einen künstlerischen oder kreativen Menschen.	○	○	○	○	○
Andere nennen mich oft einen Perfektionisten.	○	○	○	○	○
Es fällt mir schwer, mit anderen einen Kompromiss einzugehen, wenn ich überzeugt bin, dass ich Recht habe.	○	○	○	○	○
Das erste, was ich an einem neuen Ort tue, ist, Freundschaften zu schließen.	○	○	○	○	○
Ich diskutiere selten meine Probleme mit anderen Leuten.	○	○	○	○	○
Es würde mir viel Freude bereiten, teure Luxusgüter zu besitzen.	○	○	○	○	○
Ich finde es langweilig, über Philosophie zu diskutieren.	○	○	○	○	○
Ich ziehe es vor, das zu tun, was mir gerade in den Sinn kommt, anstatt an einem Plan festzuhalten.	○	○	○	○	○
Es fällt mir schwer, mich zu beherrschen, wenn Leute mich beleidigen.	○	○	○	○	○
Die meisten Leute sind aufgedrehter und dynamischer als ich es im Allgemeinen bin.	○	○	○	○	○

Ich bleibe emotionslos, selbst in Situationen, in denen die meisten Leute sehr sentimental werden.	○	○	○	○	○
Ich will, dass alle wissen, dass ich eine wichtige angesehene Person bin.	○	○	○	○	○
Ich habe Mitgefühl mit Menschen, die weniger Glück haben als ich.	○	○	○	○	○
Ich versuche, Notleidende großzügig zu unterstützen.	○	○	○	○	○
Es würde mich nicht stören, jemandem zu schaden, den ich nicht mag.	○	○	○	○	○
Man hält mich für einen hartherzigen Menschen.	○	○	○	○	○

Seite 13

PE06

	stimme überhaupt nicht zu				stimme voll zu
Es ist nicht ratsam, seine Geheimnisse preiszugeben.	○	○	○	○	○
Ich setze gerne raffinierte Manipulationen ein, um zu kriegen, was ich will.	○	○	○	○	○
Man muss wichtige Personen auf seine Seite ziehen, was auch immer es kostet.	○	○	○	○	○
Vermeide direkte Konflikte mit anderen, denn sie könnten in der Zukunft von Nutzen sein.	○	○	○	○	○
Es ist ratsam, Informationen im Hinterkopf zu behalten, die man später gegen bestimmte Personen verwenden kann.	○	○	○	○	○
Man sollte auf den richtigen Moment warten, um sich an Menschen zu rächen.	○	○	○	○	○
Manche Dinge sollte man verbergen, um seinen Ruf zu schützen.	○	○	○	○	○
Stelle sicher, dass deine Pläne dir und nicht den Anderen nutzen.	○	○	○	○	○
Die meisten Menschen können manipuliert werden.	○	○	○	○	○

PE07

	Stimme überhaupt nicht zu				stimme voll zu
Andere sehen mich als natürliche Führungsperson.	○	○	○	○	○
Viele Gruppenaktivitäten sind ohne mich ziemlich lahm.	○	○	○	○	○
Ich weiß, dass ich etwas Besonderes bin, da mir das jeder sagt.	○	○	○	○	○
Ich mag es, wichtige Personen kennenzulernen.	○	○	○	○	○
Ich bin schon mal mit berühmten Persönlichkeiten verglichen worden.	○	○	○	○	○
Ich bestehe darauf, den Respekt zu erhalten, der mir gebührt.	○	○	○	○	○
Ich hasse es, im Mittelpunkt zu stehen.	○	○	○	○	○
Ich werde verlegen, wenn mir jemand Komplimente macht.	○	○	○	○	○
Ich bin ein durchschnittlicher Mensch.	○	○	○	○	○

PE08

	stimme überhaupt nicht zu				stimme voll zu
Ich mag es, mich an Autoritäten zu rächen.	○	○	○	○	○
Eine Racheaktion muss schnell und fies sein.	○	○	○	○	○
Menschen sagen oft, dass ich außer Kontrolle bin.	○	○	○	○	○
Es stimmt, dass ich gemein sein kann.	○	○	○	○	○
Menschen bereuen es jedes Mal, wenn sie sich mit mir anlegen.	○	○	○	○	○
Ich genieße es, Sex mit Menschen zu haben, die ich kaum kenne.	○	○	○	○	○
Ich würde alles sagen, um zu bekommen, was ich will.	○	○	○	○	○
Ich vermeide gefährliche Situationen.	○	○	○	○	○
Ich bin noch nie mit dem Gesetz in Konflikt geraten.	○	○	○	○	○

Seite 14

GE01

4. Als kleines Dankeschön verlosen wir unter den Teilnehmenden 3 x 50€. Wenn Sie an dem Gewinnspiel teilnehmen möchten, tragen Sie bitte Ihre E-Mail Adresse in das untenstehende Feld ein. Sind Sie unter den Gewinnern dabei, kontaktieren wir Sie über diese E-Mail-Adresse.

☐ Ich will am Gewinnspiel teilnehmen. Ich willige ein, dass meine E-Mail-Adresse bis zur Ziehung der Gewinner gespeichert wird. Diese Einwilligung kann ich jederzeit widerrufen. Meine Angaben in dieser Befragung bleiben weiterhin anonym, meine E-Mail-Adresse wird getrennt von meinem Fragebogen gespeichert und nicht an Dritte weitergegeben.

Letzte Seite

Vielen Dank für Ihre Teilnahme!

Wir möchten uns ganz herzlich für Ihre Mithilfe bedanken.

Ihre Antworten wurden gespeichert, Sie können das Browser-Fenster nun schließen.

Möchten Sie in Zukunft an interessanten und spannenden Online-Befragungen teilnehmen?

Wir würden uns sehr freuen, wenn Sie Ihre E-Mail-Adresse für das SoSci Panel anmelden und damit wissenschaftliche Forschungsprojekte unterstützen.

E-Mail: [ ] Am Panel teilnehmen

Die Teilnahme am SoSci Panel ist freiwillig, unverbindlich und kann jederzeit widerrufen werden. Das SoSci Panel speichert Ihre E-Mail-Adresse nicht ohne Ihr Einverständnis, sendet Ihnen keine Werbung und gibt Ihre E-Mail-Adresse nicht an Dritte weiter.

Sie können das Browserfenster selbstverständlich auch schließen, ohne am SoSci Panel teilzunehmen.

Christina Daum, Leopold-Franzens-Universität Innsbruck – 2023
